# ERO1L promotes IL6/sIL6R signaling and regulates MUC16 expression to promote CA125 secretion and the metastasis of lung cancer cells

**DOI:** 10.1038/s41419-020-03067-8

**Published:** 2020-10-14

**Authors:** Yuanyuan lei, Ruochuan Zang, Zhiliang Lu, Guochao Zhang, Jianbing Huang, Chengming Liu, Zhanyu Wang, Shuangshuang Mao, Yun Che, Xinfeng Wang, Sufei Zheng, Lingling Fang, Nan Sun, Jie He

**Affiliations:** National Cancer Center/National Clinical Research Center for Cancer/Cancer Hospital, Chinese Academy of Medical Sciences and Peking Union Medical College, Beijing, China

**Keywords:** Cancer screening, Non-small-cell lung cancer, Translational research

## Abstract

The abnormal secretion of CA125, a classic tumor marker, is usually related to a poor prognosis in various tumors. Thus, this study aimed to explore the potential mechanisms that promote CA125 secretion in lung cancer. By querying the database, the gene endoplasmic reticulum oxidoreductase 1L (ERO1L) was identified and chosen as the research subject. The antibody chips were used to screen the lung cancer cell supernatant and found that the most obvious secreted protein was CA125. ERO1L was found to promote the secretion of IL6R by affecting the formation of disulfide bonds. IL6R bound to IL6 and triggered the activation of the NF-κB signaling pathway. Then, NF-κB bound to the promoter of MUC16, resulting in overexpression of MUC16. The extracellular segment of MUC16 was cleaved to form CA125, while the C terminus of MUC16 promoted the EMT phenotype and the release of IL6, forming a positive feedback pathway. In conclusion, ERO1L might affect the secretion of CA125 through the IL6 signaling pathway and form a positive feedback loop to further promote the development of lung cancer. This might expand the application scope of CA125 in lung cancer.

## Introduction

Lung cancer ranks first in morbidity and mortality^1,2^. The main cause of lung cancer-related death is the expansion of primary tumors and spreading distant metastases. At present, the most classic prognostic markers of lung cancer are protein markers, mainly secreted proteins. An abnormal increase in protein tumor markers is related to the poor prognosis of many tumors. However, many markers only play a role in indicating progression of certain disease. It is unclear why their abnormal secretion is related to the malignant progression of tumors. This secretion may be a result rather than a cause of disease progression. If the potential source that affects the secretion of these proteins can be found upstream and suppressed, maybe it may alleviate the malignant progression of the tumor to a certain extent.

The formation of intermolecular or intramolecular disulfide bonds is an important step in the process of protein folding and secretion, and is of great significance for maintaining the stability of the tertiary structure of proteins^3^. Through analyzing public databases, we found a series of genes that might affect the prognosis of lung adenocarcinoma and looked for potential-related genes that affect protein secretion. We found that the second-ranked gene ERO1L (endoplasmic reticulum oxidoreductase 1L) was an enzyme that affects the formation of disulfide bonds.

The ERO1L-encoding gene is located on human chromosome 14q22.1^4^, and can be highly expressed under hypoxic conditions^5–7^, it can also be induced by C/EPB homologous protein (CHOP or GADD153, with the gene name Ddit3) under endoplasmic reticulum stress to further activate inositol 1,4,5-triphosphate receptor, which promotes calcium release leading to apoptosis^8–11^. ERO1L, which is highly expressed under hypoxia or conditions of cell stress, can alter the expression of MHC (major histocompatibility complex) molecules in tumor cells, thereby regulating tumor immune status^12^. As a key enzyme for disulfide bond formation, it binds to flavin adenine dinucleotide and specifically recognizes the reduced protein disulfide isomerase (PDI), oxidizing it through sulfur bond conversion to participate in the folding process of the new peptide chain to form a functional secreted protein^4,5,8,13–15^. It has been reported that high expression of ERO1L in a variety of tumors including breast, gastric, esophageal, and pancreatic cancer is associated with poor prognosis^16–21^. ERO1L is included in various prognostic models of lung adenocarcinoma^22,23^. However, there are few studies on the mechanism by which ERO1L affects prognosis, and its relationship with specific secreted proteins needs further study. At present, it is only known that ERO1L can promote the secretion of VEGF in a hypoxic microenvironment and lead to increased angiogenesis. The mechanism by which ERO1L is associated with the prognosis of lung cancer and the specific types of secreted proteins involved need to be explored.

Here, we explored the mechanisms by which ERO1L affects the prognosis of lung adenocarcinoma, and extensively screened the secreted proteins affected by it. We found that ERO1L promoted the secretion of the classic tumor marker CA125 through the IL6 signaling pathway and formed a positive feedback pathway.

## Results

### Screening of related genes that simultaneously affect lung cancer prognosis and protein secretion

First, we queried the GEPIA database to screen the genes that are most relevant to the prognosis of lung adenocarcinoma. The top 20 genes most significantly associated with overall survival were shown in Table S1. Then we analyzed the biological functions of these 20 genes by using the GeneCards database to identify genes that may affect the protein secretion. The protein encoded by the second-ranked gene ERO1L is an enzyme related to the synthesis of disulfide bonds. Therefore, we hypothesized that this gene was likely to be a key gene that affects both the prognosis of lung cancer and the secretion of tumor markers.

**Fig. 1:**
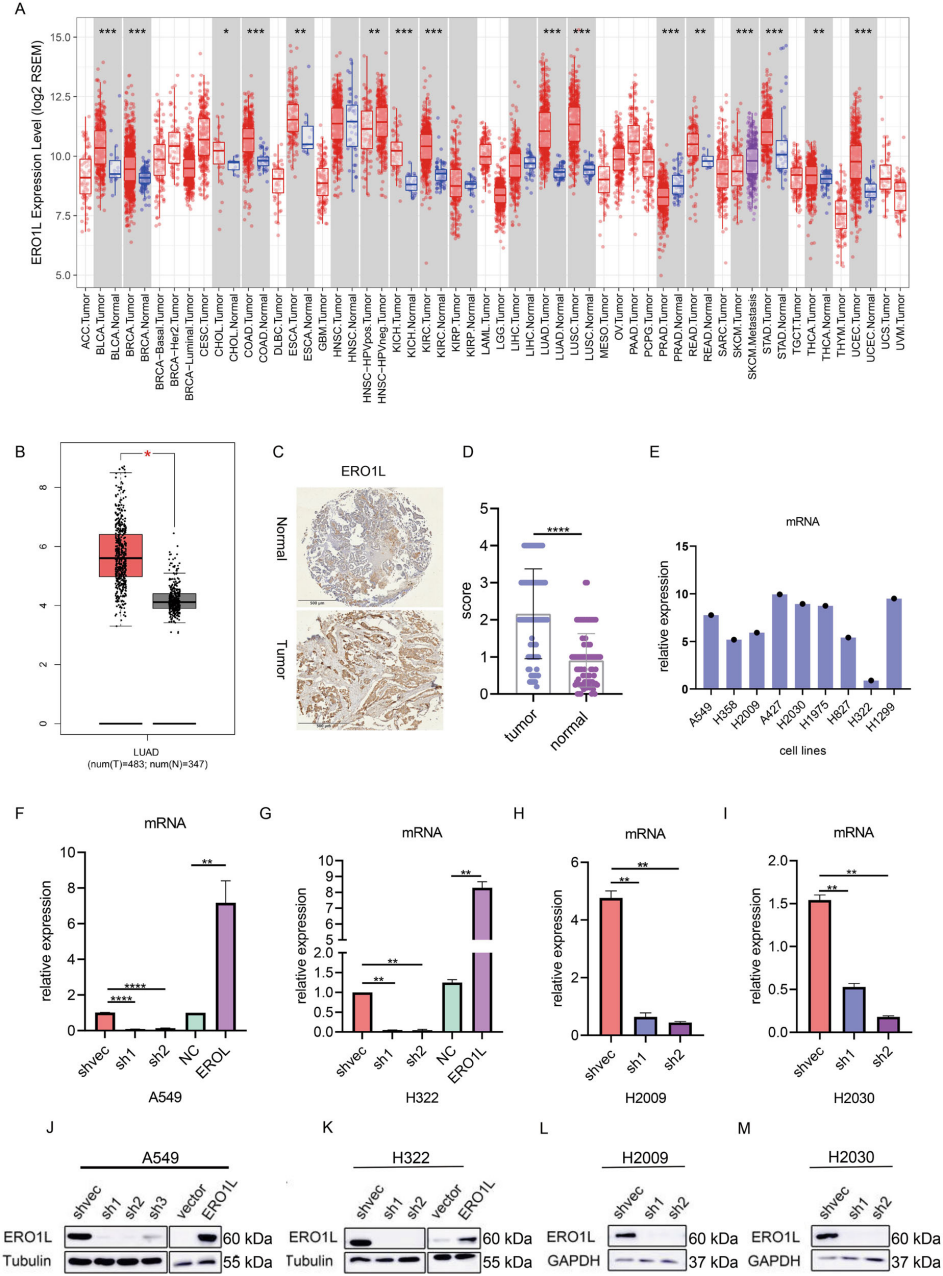
ERO1L is highly expressed in lung cancer cell lines and tissues. **A** Expression of ERO1L in different tumor tissues in the TIMER database. **B** Expression of ERO1L in GEPIA lung adenocarcinoma samples. **C** IHC showing the expression of representative ERO1L. The above figure shows expression in adjacent normal tissues, followed by expression in tumor tissues. **D** Expression of ERO1L displayed on tissue chips of 80 pairs of cancer and adjacent tissues. **E** The expression of ERO1L in different lung adenocarcinoma cell lines in the CCLE database. **F**–**M** Western blotting and PCR results showing ERO1L overexpression and knockdown efficiency in four cell lines. Data are presented as the mean ± SD, *n* = 3. Student’s *t*-test was used to analyze the results; *p < 0.05, **p < 0.01, ***p < 0.001.

We first analyzed the expression of ERO1L in the GEPIA database. The expression level of ERO1L in tumor tissues was indeed higher than that in normal tissues (Fig. 1A, B). Besides, we queried the TIMER database again and found that ERO1L was overexpressed in many other tumors (Fig. 1A). To further verify the expression of ERO1L, we performed immune-histochemical detection on tissue chips of 80 patient with lung adenocarcinoma. The standard image was shown in Fig. 1C. The results showed that the expression of ERO1L in cancerous tissues was indeed much higher than that in adjacent tissues (Fig. 1D). To facilitate subsequent in vitro experiments, we searched the Cancer Cell Line Encyclopedia (CCLE) database for the expression of ERO1L in lung adenocarcinoma-related cell lines (Fig. 1E). Combining the expression level and the generality of the cell line use, we selected four cell lines to construct stable transgenic cell lines. Among them, we constructed ERO1L overexpression and knockdown strains in A549 and H322, and only knocked down ERO1L in H2009 and H2030 (Fig. 1F–M).

### Cell culture supernatants from cell lines overexpressing ERO1L

**Fig. 2:**
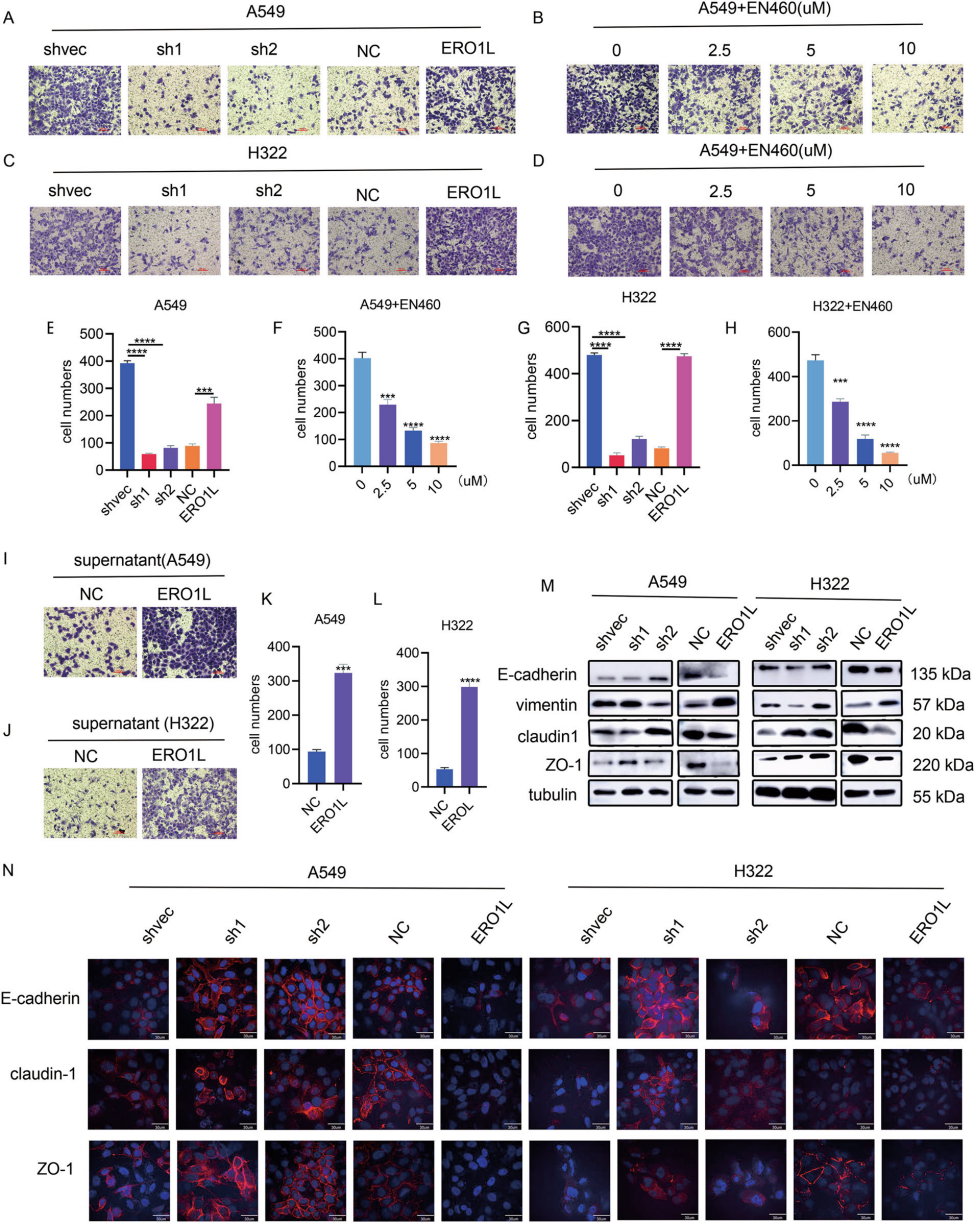
ERO1L in the cell supernatant promotes the metastasis of lung cancer cell lines. **A**, **C** Effects of overexpression or knockdown of ERO1L on the migration ability of wild-type cell lines A549 and H322. **B**, **D** Effects of different concentrations of the ERO1L inhibitor EN460 on the migration ability of wild-type cell lines A549 and H322. **E**–**H** Number of migrated cells. **I**–**L** Effect of cell supernatant from ERO1L-overexpressing cells on the migration ability of wild-type cell lines A549 and H322. **M** Western blots showing changes in the EMT-related indicators in A549 and H322 cells after overexpression or knockdown of ERO1L. **N** Immunofluorescence results showing changes in EMT-related three surface proteins. Data are presented as the mean ± SD, *n* = 3. Student’s *t*-test was used to analyze the results; *p < 0.05, **p < 0.01, ***p < 0.001.

To explore the function of ERO1L in lung adenocarcinoma, we used the abovementioned stable transfected cell lines to perform in vitro experiments. The results showed that overexpression of ERO1L promoted the migration of tumor cells A549 and H322, whereas knockdown n of ERO1L inhibited migration (Fig. 2A, C, E, G). To further verify the migration-promoting function of ERO1L, we purchased EN460, an inhibitor of ERO1L, and set a concentration gradient, 0, 2.5, 5, and 10 µM. Within this concentration range, the expression level of ERO1L was downregulated and cell viability was not significantly reduced (Fig. S2A–C). The results showed that the migration capacity of A549 and H322 cells was indeed reduced to a certain extent after treatment with different concentrations of EN460 (Fig. 2B, D, F, H).

We mentioned earlier that ERO1L was likely to cause the secretion of some secreted proteins. We wondered whether this migration-promoting function of ERO1L was also achieved indirectly by cytokine secretion. We therefore collected cell supernatants from ERO1L-overexpressing cell lines and cocultured these supernatants with wild-type A549 and H322 cells. The results showed that the migration ability of A549 and H322 cells cocultured with the cell supernatant was significantly enhanced compared to that of non-cocultured cells (Fig. 2I–L). Considering that cancer cells with increased migration capacity usually undergo EMT (epithelial to mesenchymal transition), we analyzed several EMT markers by western blotting. The results showed that in ERO1L knockdown cell lines, the interstitial marker vimentin was significantly reduced while the epithelial markers E-cadherin, ZO-1, and claudin-1 were significantly increased; the opposite was true for ERO1L-overexpressing cell lines (Fig. 2M). In addition, we further verified the expression levels of E-cadherin, ZO-1, and claudin-1 on the surface of the three cell lines by immunofluorescence, and the results were consistent with the western blot results (Fig. 2N). The above results indicated that the culture supernatant of ERO1L-overexpressing cell lines could promote tumor cell migration.

### ERO1L significantly activates chemotaxis-related signaling pathways and promotes the secretion of a series of cytokines

**Fig. 3:**
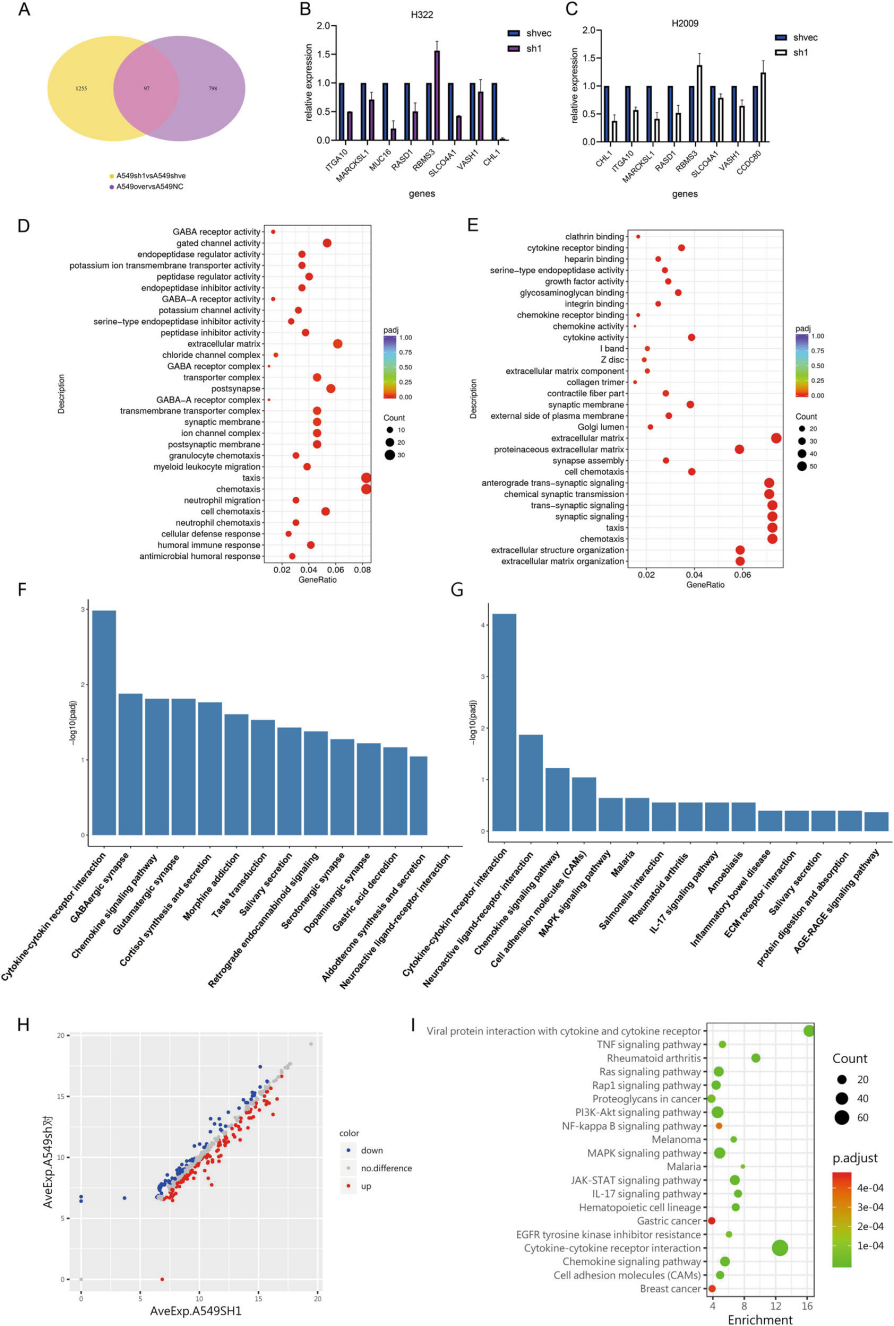
Chemotaxis-related signaling pathways are activated and accompanied by the secretion of a series of cytokines. **A** The Venn diagram shows the number of genes co-upregulated or downregulated after ERO1L knockdown or overexpression. **B**, **C** Verification of RNA-seq results. Eight genes were randomly selected, and their expression levels were verified by PCR. The results were consistent with the sequencing results in the two cell lines. **D**, **E** Bubble chart shows the more obvious signaling pathways affected by ERO1L in the two cell lines. **F**, **G** Histogram showing enriched signaling pathways in KEGG. **H** The bubble chart showing the enrichment of differentially expressed proteins in the cell supernatant. **I** Scatter plot showing the distribution of 213 differentially expressed proteins enriched in cell supernatants. Data are presented as the mean ± SD, *n* = 3. Student’s *t*-test was used to analyze the results; *p < 0.05, **p < 0.01, ***p < 0.001.

To further explore the mechanism by which ERO1L promotes metastasis, we performed transcriptome sequencing of stably transfected cell lines, including A549 cells with overexpression or knockdown of ERO1L and H322 cells with knockdown of ERO1L. For the A549 cell line, 798 genes were upregulated in the ERO1L overexpression group and 1255 genes were downregulated in the ERO1L knockdown group, with 97 differentially expressed in common (Fig. 3A). Among them, MUC16 (encoding the classic protein CA125) had the most obvious difference. We randomly selected eight genes in the cell lines H322 and H2009 to verify the expression levels by PCR. We found that the expression trend of these eight genes was consistent with that in the RNA-seq data (Fig. 3B, C), indicating that the transcriptome sequencing data are accurate. The differentially expressed genes were then subjected to functional enrichment analysis. The bubble chart showed that chemotaxis-related functions were enriched in the two groups (Fig. 3D, E), and the histogram of KEGG pathway enrichment showed that the cytokine-cytokine interaction signal pathway was also enriched (Fig. 3F, G). These results suggested that the ability of ERO1L to promote metastasis might indeed be achieved by certain factors in the cell supernatant.

Next, to determine which component of the cell supernatant promoted tumor cell metastasis, we collected the cell culture supernatant of the A549 cell line with knockdown ERO1L and used an antibody chip containing 440 factors to detect differentially expressed proteins. We found a total of 213 differential proteins (Fig. 3H). Similarly, we performed functional enrichment and KEGG pathway enrichment analyses of these differentially expressed proteins. The enrichment results were consistent with those form the RNA-seq results, which revealed significant changes in pathways related to chemotaxis and cytokine interactions (Fig. 3I).

### ERO1L regulates IL6R and CA125 secretion through disulfide bonds

**Fig. 4:**
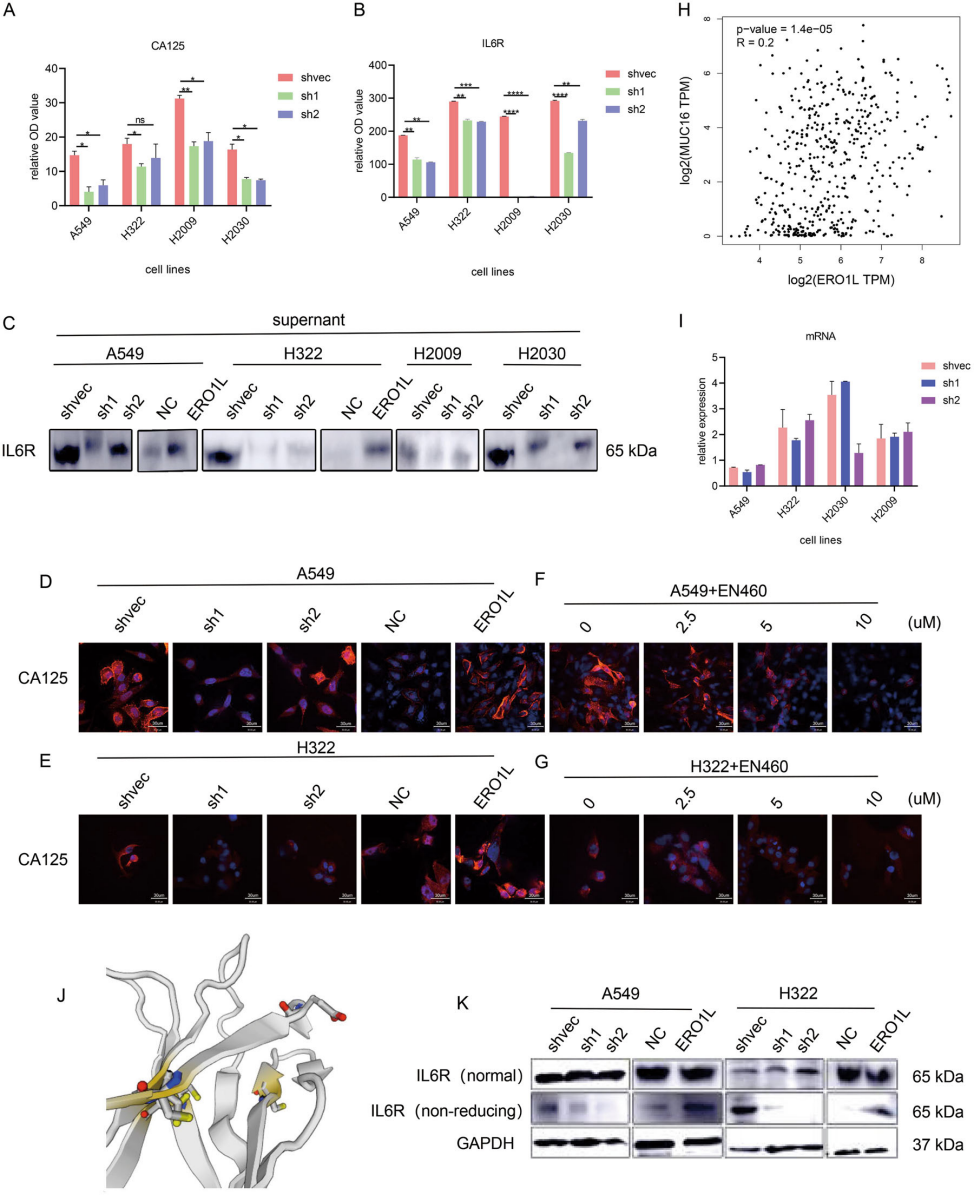
ERO1L regulates IL6R and CA125 secretion through disulfide bonds. **A**, **C** Immunofluorescence image showing changes in CA125 expression in different stable cell lines. **B**, **D** Immunofluorescence results showed changes in CA125 expression after treatment with different concentrations of ERO1L inhibitor. **E**, **F** Changes in CA125 and IL6R in culture supernatants of different stable cell lines were verified by ELISA experiments. **G** Changes in the mRNA levels of IL6R in stably transfected cell lines were verified by PCR experiments. **H** Analysis of the correlation between ERO1L and MUC16 in the GEPIA database. **I** Changes in IL6R secretion in supernatants of different stably transfected cells were verified by western blotting. **J** Three-dimensional structure of IL6R and possible regions (yellow) for disulfide bond formation. **K** Western blots under reduced and nonreduced conditions showing changes in IL6R protein. Data are presented as the mean ± SD, *n* = 3. Student’s *t*-test was used to analyze the results; *p < 0.05, **p < 0.01, ***p < 0.001.

We further screened the 213 differentially expressed genes selected by ELISA. First, we eliminated possible noise signals (fluorescence values less than 1000) and the top two proteins were CA125 and IL6R (Fig. 4A, B). For IL6R, western blots confirmed that the level of IL6R in the cell supernatant was consistent with that in the ELISA (Fig. 4C). Since CA125 is encoded by the MUC16 gene and the protein has a particularly high molecular weight, it was difficult to verify the protein expression in western bot experiments. We performed immunofluorescence staining to further verify the expression of CA125. The results confirmed that overexpression of ERO1L increased the expression of CA125 protein (Fig. 4D, E). The expression of CA125 in cells treated with different concentrations of ERO1L inhibitors also decreased in a dose-dependent manner (Fig. 4F, G). Then, we wondered which of the two proteins was affected directly by ERO1L. As mentioned earlier, ERO1L could affect protein secretion and not protein synthesis, that is, there is no change at the mRNA level. However, we noticed that MUC16 was significantly upregulated at the mRNA level (Fig. 3B, C). GEPIA database analysis also found that ERO1L was positively correlated with MUC16 mRNA expression (Fig. 4H). Although there was an increase in IL6R protein expression and secretion, IL6R mRNA expression was not significantly upregulated (Fig. 4I). Moreover, when we analyzed the crystal structure of IL6R, we found that there were indeed two disulfide bonds (Fig. 4J); however, the molecular weight of MUC16 is particularly high, and the current crystal structure has not been resolved. We speculated that the increased secretion of IL6R might be directly affected by ERO1L, while the increased secretion of CA125 might be indirectly achieved by the increase in mRNA. To verify that ERO1L affected the secretion of IL6R by affecting the formation of disulfide bonds of IL6R, western blotting was performed on different samples, and the expression level of IL6R in ERO1L overexpressing or ERO1L knockdown cell lines remained unchanged under reducing conditions, while it was significantly changed under nonreducing conditions (Fig. 4K).

### IL6R promotes MUC16 expression and CA125 secretion by activating the NF-κB pathway

**Fig. 5:**
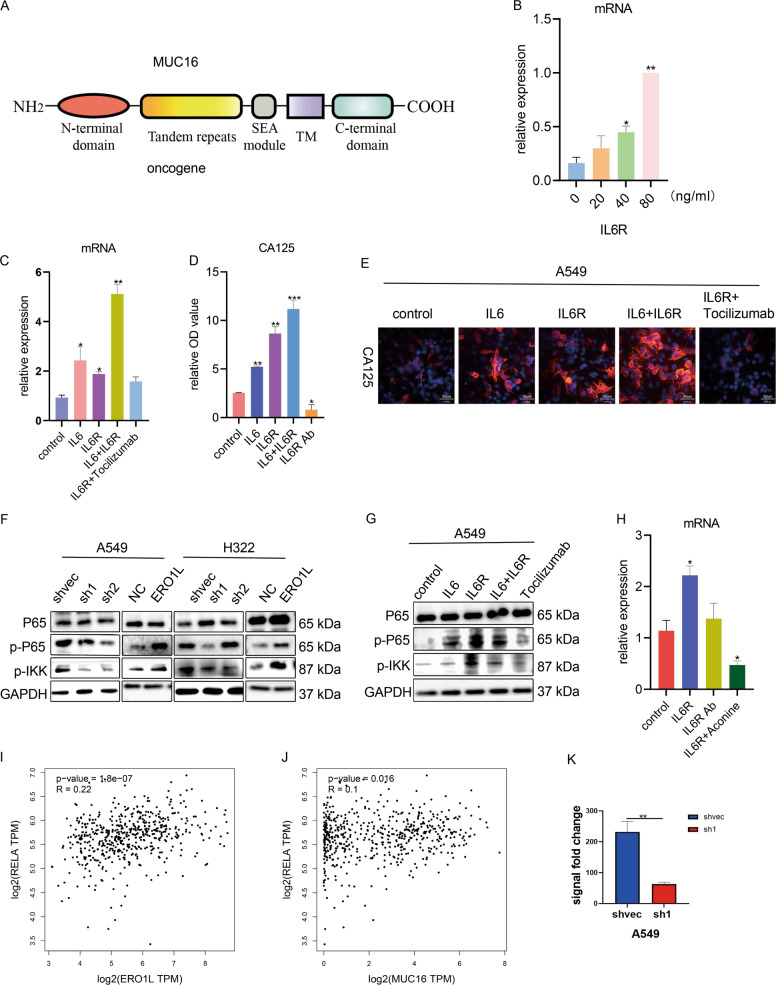
IL6R can activate the NF-kB signaling pathway. **A** Illustration of the full-length MUC16 protein. The entire protein is divided into the extracellular N terminus, intermediate transmembrane repeats, and intracellular C terminus. **B** Changes in CA125 in wild-type A549 cells after treatment with IL6R and its related factors. **C** PCR results showed changes in MUC16 mRNA of wild-type A549 cells treated with different concentrations of IL6R. **D** PCR results showed changes in MUC16 mRNA levels after treatment of wild-type A549 with IL6R and its related factors. **E** ELISA results showed changes in the secretion of CA125 in cell supernatants of wild-type A549 cells after the treatment with IL6R and its related factors. **F** Western blots showing changes in the NF-κB signaling pathway after treatment of wild-type A549 cells with different factors. **G** Changes in the NF-κB signaling pathway after ERO1L overexpression or knockdown. **H** Changes in MUC16 mRNA in wild-type A549 cells after treatment with IL6R monoclonal antibody and NF-κB inhibitor. **I** Coimmunoprecipitation experiments showed changes in the binding ability of NF-κB to the MUC16 promoter after ERO1L knockdown. **J**, **K** The GEPIA database showed correlations among NF-kB, MUC16, and ERO1L. Data are presented as the mean ± SD, *n* = 3. Student’s *t*-test was used to analyze the results; *p < 0.05, **p < 0.01, ***p < 0.001.

The full length of MUC16 gene is divided into three parts; the extracellular N-terminal region (later converted to CA125), the transmembrane region and the intracellular C terminus (promoting the occurrence and development of multiple tumors)^24–26^ (Fig. 5A). The IL6 signaling pathway is a classic pathway that regulates the expression and entry of multiple transcription factors into the nucleus. We therefore wondered whether the secretion of IL6R caused the upregulation of MUC16. When we cocultured the A549 cells with IL6R, we observed that MUC16 mRNA was significantly upregulated in a concentration-dependent manner (Fig. 5B). This upregulation effect was more obvious after increasing the concentration of IL6, but this effect weakened after adding the monoclonal antibody tocilizumab to IL6R (Fig. 5C). Since CA125 is encoded by MUC16, we detected the secretion of CA125 in A549 cells before and after treatment with IL6R or its inhibitors by ELISA. The results showed that IL6R promoted the secretion of CA125, while tocilizumab inhibited its secretion (Fig. 5D). In addition, the immunofluorescence results were consistent with the PCR and ELISA results; that is, IL6R significantly promoted the expression of CA125 (Fig. 5E).

Since IL6R can cause the activation of multiple signaling pathways, we wandered how IL6R upregulated the expression of MUC16. By consulting the literature, we found that the NF-κB molecule could bind to the promoter region of MUC16 and promote its expression^27^. We therefore wondered whether IL6R could also activate NF-κB signaling, leading to upregulation of MUC16. Western blotting showed that the NF-κB pathway was indeed activated after overexpression of ERO1L in stable transfected cell lines, and knockdown of ERO1L inhibited the activity of the pathway (Fig. 5F). At the same time, we showed that when IL6R was added to A549 cells, the NF-κB signaling pathway was also significantly activated. When IL6 was present, the activation effect was superimposed, but this phenomenon was inhibited by tocilizumab (Fig. 5G). Moreover, the ability of IL6R to promote MUC16 expression was significantly reduced in the presence of the NF-κB inhibitor aconine (Fig. 5H). We also found that the expression of ERO1L and NF-κB was positively correlated through the analysis of the GEPIA database (Fig. 5I), and the expression of MUC16 was also positively correlated with the expression of NF-κB (Fig. 5J). Finally, we verified by chromatin immunoprecipitation experiments whether the NF-κB molecule could bind to the MUC16 promoter. First, we used the LASAGNA-Search database to predict the types and binding sites of transcription factors that the MUC16 promoter region might bind. NF-κB was included in the results. We designed chip primers based on the predicted binding sequences, and used them in subsequent coimmunoprecipitation experiments (primer sequences are shown in the Supplementary materials). The chip results showed that knockdown of ERO1L significantly reduced the ability of NF-κB to bind to the MUC16 promoter sequence (Fig. 5K).

### MUC16-C enhances the IL6 signaling pathway to form a positive feedback loop

**Fig. 6:**
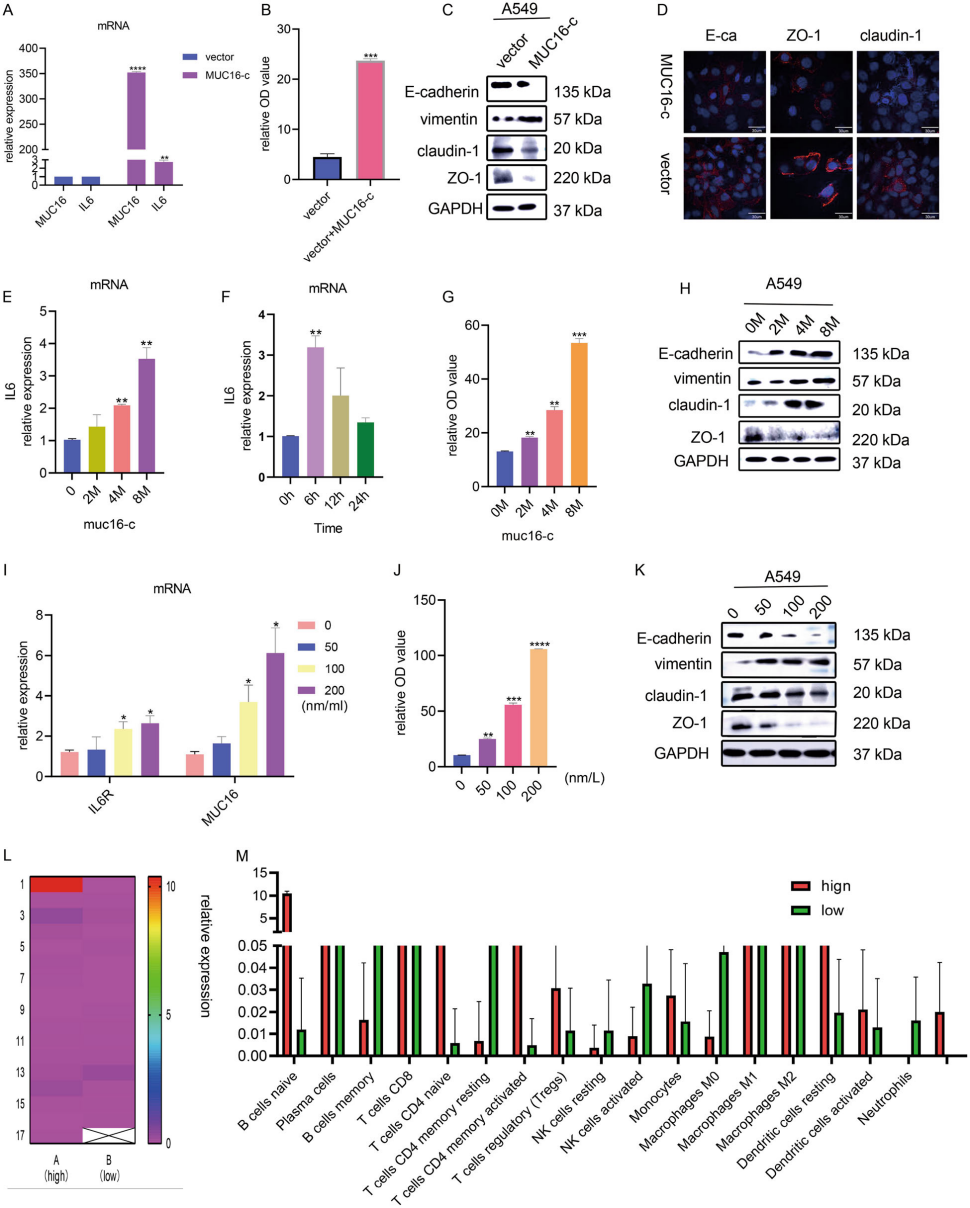
MUC16-C enhances the IL6 signaling pathway via a positive feedback loop. **A** PCR experiments verified the efficiency of MUC16-C overexpression. **B** ELISA results showed changes in IL6 secretion in cell supernatants after MUC16 overexpression. **C** Changes in EMT-related indicators after MUC16-C overexpression. **D** Immunofluorescence assay showed changes in three surface proteins in EMT indicators. **E** Expression of IL6 in wild-type A549 cells after coculture with MUC16 polypeptide at different times. **F** Expression of IL6 in wild-type A549 cells cocultured with different concentrations of MUC16 peptide. **G** ELISA experiments showed changes in IL6 content in cell supernatants after coculture of A549 cells with different concentrations of MUC16 peptide. **H**, **K** Changes in EMT indicators after coculture. **I** PCR results showed the effect of EGF on the mRNA of MUC16 and IL6. **J** ELISA experiments showed changes in IL6 content in cell supernatants. **L**, **M** Bioinformatics analysis showed the recruitment of leukocytes by ERO1L. The numbers in the L chart correspond to the names of the abscissas in the M chart. Data are presented as the mean ± SD, *n* = 3. Student’s *t*-test was used to analyze the results; *p < 0.05, **p < 0.01, ***p < 0.001.

IL6R requires the assistance of IL6 to function. Some studies have reported that the C terminus of MUC16 (MUC16-C) can promote the secretion of IL6^26^. Through the ELISA method, we found that the culture supernatant of A549 cell line did have a certain amount of IL6 (Fig. S3D–F). We wondered whether this mechanism was also found in lung cancer cell lines and verified this conjecture in three ways. First, we constructed an MUC16-C overexpression plasmid and transfected it into A549 cell line. The transfection efficiency was shown in Fig. 6A. With the overexpression of MUC16-C we found that IL6 increased in mRNA levels (Fig. 6A). At the same time, the amount of the secreted protein IL6 in the cell supernatant also increased (Fig. 6B). Second, we synthesized a peptide fragment of MUC16-C (see Supplementary materials for the sequence) and cocultured MUC16-C with A549 at different times and doses. It was found that the MUC16-C peptide significantly promoted the upregulation of IL6, and the effect was dose-dependent (Fig. 6E) but reached a peak at 6 h (Fig. 6F). ELISA experiments demonstrated the similar results (Fig. 6G). Third, we added the cytokine EGF. According to reports, EGF can promote the transfer of MUC16-C into the cytoplasm and play a tumor-promoting role^26^. We speculated that EGF could also promote IL6 secretion. When different concentrations of EGF were cocultured with A549 cells, MUC16-C did increase to different degrees and IL6 also increased in a concentration-dependent manner (Fig. 6I). Similarly, IL6 was also elevated in the cell culture supernatant (Fig. 6J).

As mentioned earlier, the migration capacity of lung cancer cell lines overexpressing ERO1L was significantly enhanced and was accompanied by an increase in EMT-related markers. MUC16-C has also been reported to promotes tumorigenesis. We wondered whether this migration-promoting ability of ERO1L was determined by this characteristic of MUC16-C. Therefore, we analyzed whether the EMT-related markers changed in the above three cases. It showed that under the conditions of MUC16-C overexpression, MUC16-C polypeptide coculture, or EGF coculture, the EMT-related markers did change in the direction that promoted cell migration (Fig. 6C, H, K). In the case of MUC16 overexpression, three indicators related to cell adhesion were also confirmed to be significantly downregulated by immunofluorescence (Fig. 6D).

Overexpression of ERO1L might promote the secretion of IL6R, but the function of IL6R requires the assistance of IL6. Although MUC16-C could promote the secretion of IL6, the effect is not dramatic, and the cells that secrete the most IL6 in the microenvironment are not tumor cells but white blood cells. We speculated that ERO1L also recruited white blood cells as a continuous source of IL6. To prove this conjecture, we downloaded the related data set (GSE30219) from the GEO database and sorted the samples into two groups according to the expression level of ERO1L; then, we analyzed the white blood cells in the two groups by using CIBERSORT. The initial B cells were significantly enriched in cells with high expression of ERO1L (Fig. 6L, M). We extracted B cells from the tissues of five lung cancer patients, the clinical information was shown in Table S2. IL6 was detected from the culture supernatant of B cells by ELISA. The results showed that B cells were indeed the important cells that secreted IL6 (Fig. S3A–E). The above results together showed that ERO1L simultaneously promoted the secretion of IL6 and IL6R and forms a positive feedback loop with MUC16, which promoted the migration of lung adenocarcinoma cells and the release of the classic marker CA125.

### ERO1L promotes tumor metastasis in vivo and cause poor prognosis

**Fig. 7:**
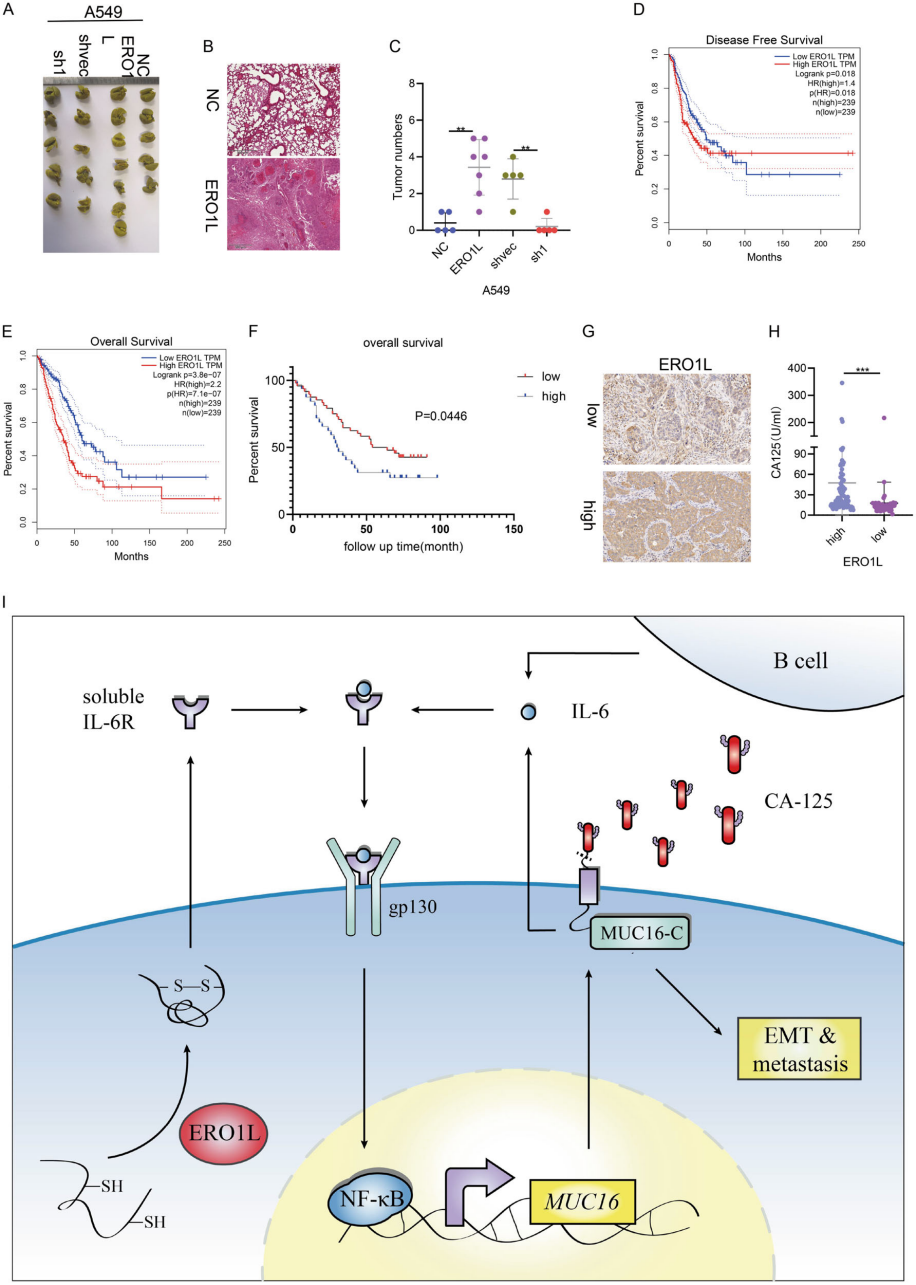
ERO1L promotes tumor metastasis in vivo and is related to poor prognosis. **A**, **B** Relationship between ERO1L expression and disease-free survival and overall survival of lung adenocarcinoma patients in the GEPIA database. **C** Relationship between ERO1L expression and overall survival validated by tissue microarrays containing 80 specimens from our laboratory. **D** Representative pictures of lung colonization experiments. **E** Representative HE staining of the lung tissue. **F** Statistics of the number of metastases in different groups. **G** IHC images of representative lung cancer tissues. **H** Scatter plot showing CA125 content in the ERO1L high and low expression groups. **I** A schematic model of REO1L functions in NSCLC. Data are presented as the mean ± SD, *n* = 3. Student’s *t*-test was used to analyze the results; *p < 0.05, **p < 0.01, ***p < 0.001.

We observed the metastasis-promoting ability of ERO1L in vivo through a lung colonization model generated by tail vein injection of A549 cells. The lung colonization of different cell lines was shown in Fig. 7A, B. In vivo experiments showed that overexpression of ERO1L promoted the metastasis of lung cancer cells, while knockdown of ERO1L inhibited metastasis (Fig. 7C).

Next, we queried the GEPIA database and found that the disease-free survival rate and overall survival rate of 239 patients with high expression of ERO1L were much lower than those of 239 patients with low expression of ERO1L (Fig. 7D, E). At the same time, we also analyzed the expression levels of ERO1L in 80 lung adenocarcinoma tissue chips in the laboratory. Among them, 48 of 80 tissues showed high expression of ERO1L, and 32 showed low expression of ERO1L. Follow-up patient information revealed that the prognosis results of these patients were similar to those of the database, that is, high expression of ERO1L led to a poorer prognosis than low expression, and the difference was statistically significant (Fig. 7F).

In vitro experiments confirmed that ERO1L promoted the secretion of the classic tumor marker CA125. We collected tissue samples from 133 patients with lung adenocarcinoma who had undergone surgery and had previously detected CA125. By examining the expression of ERO1L in tissues (representative pictures are shown in Fig. 7G), we found that CA125 levels in peripheral blood did have a clear correlation with the expression of ERO1L and the levels of secreted CA125 in patient with high expression of ERO1L were higher than those with low expression (Fig. 7H).

## Discussion

In this article, we focused on the regulatory factors that affect the secretion of tumor markers. We found that ERO1L not only significantly affected the prognosis of lung adenocarcinoma, but also promoted the secretion of the classic tumor marker CA125 in a positive feedback form (Fig. 7I).

ERO1L causes the folding and formation of disulfide bonds of proteins in the endoplasmic reticulum by interacting with PDI^28^. The formation of disulfide bonds must have a suitable oxygen environment and ERO1L is a major producer of hydrogen peroxide (H2O2) in the ER lumen^29,30^, while the imbalance of oxygen content is closely related to endoplasmic reticulum stress. Therefore, ERO1L also affects the endoplasmic reticulum stress response. In recent years, the study of the correlation between endoplasmic reticulum stress and tumors has received increasing attention. However, little research has been done on the relationship between ERO1L and tumors, and the existing research was limited to prognostic correlations^31,32^. In our study, ERO1L was closely related to the prognosis of lung adenocarcinoma which was consistent with literature reports^33^. The use of multiple markers for diagnosis can avoid the shortcomings of a single marker. However, the current multi-marker diagnosis methods are mostly based on the combination of various indicators in peripheral blood and the specificity of blood markers is not high enough. Compared with blood markers, various histochemical indexes of tissues are relatively stable and less susceptible to physiological conditions. Because ERO1L has the potential to affect the secretion of multiple tumor markers, and at the same time is a strong prognostic factor related to lung adenocarcinoma, combining traditional blood markers and the expression of ERO1L in tissues to establish a diagnostic and prognostic model may have a good effect.

As mentioned earlier, ERO1L promoted the migration of lung cancer cell lines, and this promotion effect was achieved indirectly through the MUC16-C. The full-length MUC16 protein has a particularly high molecular weight, and it can promote the occurrence and development of many tumors^25,34–44^. However, the full-length protein is usually cut into three functional fragments after synthesis^45^. According to the literature, the C-terminal region of MUC16 is mainly responsible for tumorigenicity^46,47^, which is consistent with our report. Based on the strong tumorigenicity of MUC16, a variety of treatment methods such as monoclonal antibodies^48,49^ and fusion protein^50^ have been developed for MUC16^47^. However, the current treatment strategies are not effective. On the one hand, full-length MUC16 is usually dissociated into three fragments after synthesis, and the extracellular fragment gradually evolves into the secreted protein CA125. The therapeutic monoclonal antibody is generally effective in the extracellular region, and the dissociation of the extracellular region leads to the loss of target; on the other hand, the MUC16 gene fragment is relatively long and has a high mutation rate^37,44^. Some mutant strains can promote MUC16 expression^24^ and even have a higher tumorigenic effect than that of the original target change^39,51^. We confirmed that ERO1L was a key factor upstream of MUC16 that significantly affected MUC16 expression. In vitro tests confirmed that EN460, the inhibitor of ERO1L, had a clear inhibitory effect on the function of the gene itself, but this inhibitor cannot be used for in vivo studies at present. Further optimizing the in vivo inhibitory function of EN460 or combining drugs targeting MUC16 will likely improve the therapeutic effect of tumors.

In addition, we found that in the tissues with high expression of ERO1L, multiple immune cells accumulated, which might be related to ERO1L’s ability to promote the secretion of multiple cytokines. Therefore, we speculated that ERO1L might also play an important role in the regulation of the lung cancer microenvironment though processes such as immunity, inflammation, and angiogenesis. CA125, a downstream molecule of ERO1L, might be a good indicator for evaluating the efficacy of immunotherapy, which deserves further exploration.

In conclusion, ERO1L plays an important role in the development of lung cancer. First, ERO1L regulates the expression of MUC16 through the secretion of cytokines and promotes the secretion of the classic tumor marker CA125, which presents new ideas for therapy targeting MUC16 and for the use of multiple tumor markers for diagnosis. Second, ERO1L can recruit a large number of white blood cells, and regulate the expression of MHC molecules^12^, which can expand the application of CA125 in the diagnosis and treatment of lung cancer to areas such as immunotherapy.

## Materials and methods

### Patients and tumor specimens

Paraffin-embedded tissues sections for immunohistochemistry studies were obtained with the informed consent of patients with NSCLC who underwent radical resections in the Department of Thoracic Surgery of the Cancer Hospital of the Chinese Academy of Medical Sciences from 2008 to 2013. The peripheral blood samples used for ELISA were obtained from patients who underwent surgery in the Department of Thoracic Surgery of the Cancer Hospital of the Chinese Academy of Medical Sciences in 2018. Ethical approval was granted by the Committee for the Ethics Review of Research Involving Human Subjects of the Cancer Hospital of the Chinese Academy of Medical Sciences.

### In vivo animal experiments

Female athymic BALB/c nude mice and NOD/SCID mice (aged 4–5 weeks) were used for the experiments. The subcutaneous tumor growth and lung colonization assays are described in the Supplemental Experimental Procedures. The animal studies were approved by the Animal Care and Use Committee of the Cancer Hospital of the Chinese Academy of Medical Sciences.

### Statistical analysis

Statistical analysis was performed using GraphPad Prism 8.0. Comparisons were performed using Student’s *t*-test (two-tailed) and Pearson correlation analysis. All data were presented as the mean ± standard deviation. Overall survival was estimated using the Kaplan–Meier method. Differences with *p* values < 0.05 were considered significant.

For more details regarding the materials and methods, please refer to the Supplementary Materials.

## Supplementary information

supplementary figure legends

figure S1

figure S2

figure S3

supplementary
