# Supplementary material for: ERO1L promotes IL6/sIL6R signaling and regulates MUC16 expression to promote CA125 secretion and the metastasis of lung cancer cells
Source: Cell Death Dis. 2020 Oct 14;11(10):853. doi: 10.1038/s41419-020-03067-8 (PMC7560734; doi:10.1038/s41419-020-03067-8)
Supplement: Supplementary file 1 — supplementary figure legends [file 41419_2020_3067_MOESM1_ESM.docx]

**Figure S1 ERO1L has no effect on proliferation and apoptosis of lung cancer cell lines.** (**A-D**) Cell proliferation curve of A549 and H322 cells with ERO1L knockdown or overexpression. CCK8 assay was performed. (**E-F**) Cisplatin-induced apoptosis in two cell lines after knockdown or overexpression of ERO1L.

**Figure S2 Determination of the optimum concentration of EN460.** (**A**) Western blots experiments showed the inhibition of ERO1L gene by different concentrations of EN460. (**B-C**) CCK8 experiment showed the effect of maximum inhibitory concentration on proliferation of A549 and H322 cells.

**Figure S3 Basic IL6 secretion levels in lung cancer tissues and cell lines.** (**A-B**) Verification of the efficiency by flow cytometry for sorting B cells by magnetic bead method. CD45 and CD19 double positive cells were considered as B cells, and it could be seen that the positive rate of B cells after magnetic bead sorting reached about 90%. (**C**) The number of B cells sorted out from the five tumor specimens was displayed. (**D**) Standard curve of IL6 by ELISA. (**E**) Scatterplot showing the secretion of IL6 in five tumor tissues detected by ELISA. Before the ELISA detection, B cells isolated from each specimen were placed in 1 ml medium for 48 h. (**F**) Histogram showing the basic secretion of IL6 in A549 cell line detected by ELISA.

Table S1 the top twenty genes most significantly associated with overall survival

Table S2 Clinical information of five lung cancer patients used to extract B cells

Table S3 Possible binding sites of NF-kB and MUC16 promoter

Table S4 Primers used for real-time PCR

Table S5 shRNA target sequences for ERO1L
