# Supplementary figures and images for: ERO1L promotes IL6/sIL6R signaling and regulates MUC16 expression to promote CA125 secretion and the metastasis of lung cancer cells
Source: Cell Death Dis. 2020 Oct 14;11(10):853. doi: 10.1038/s41419-020-03067-8 (PMC7560734; doi:10.1038/s41419-020-03067-8)

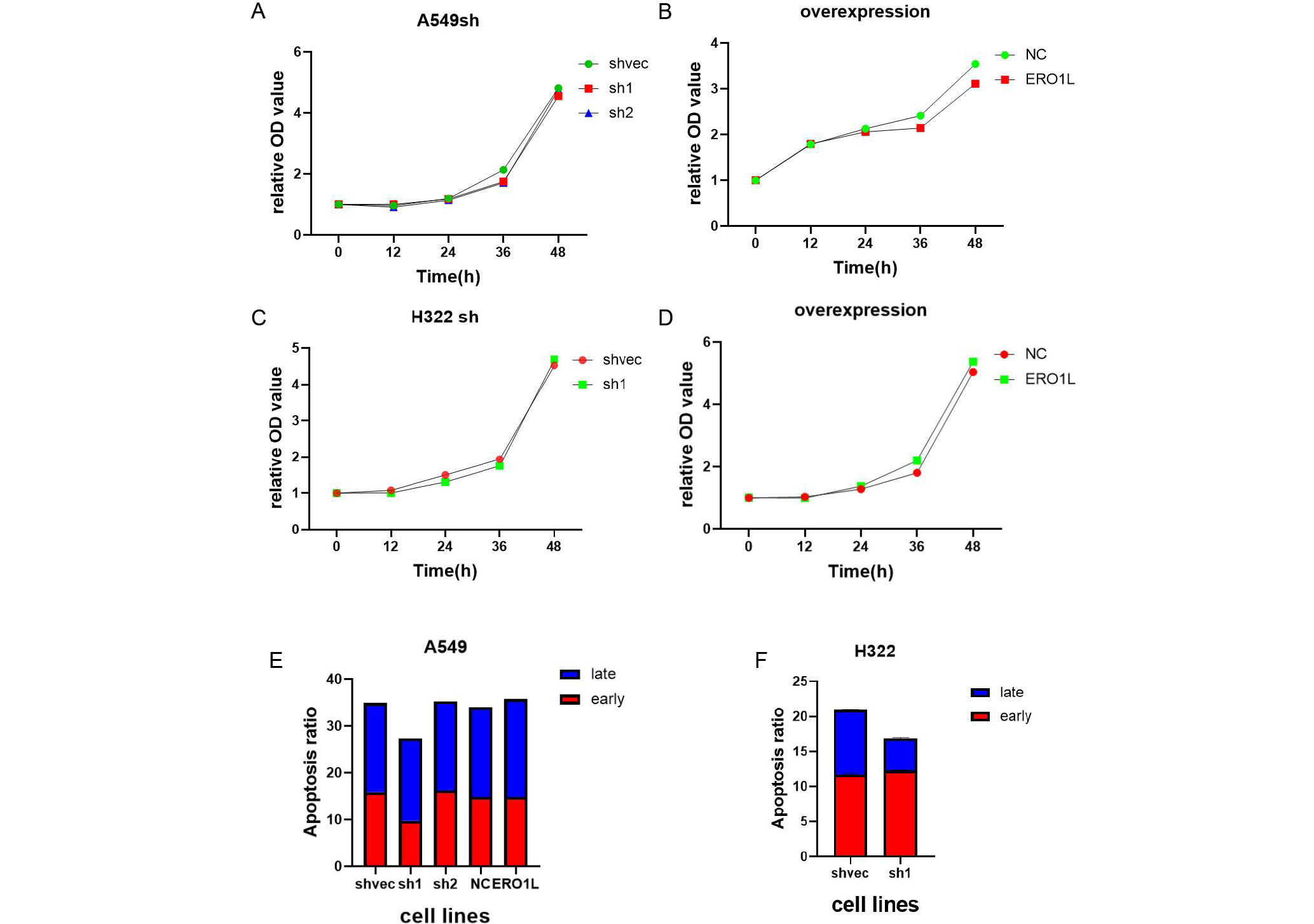

Supplement: Supplementary file 2 — figure S1 [file 41419_2020_3067_MOESM2_ESM.tif]

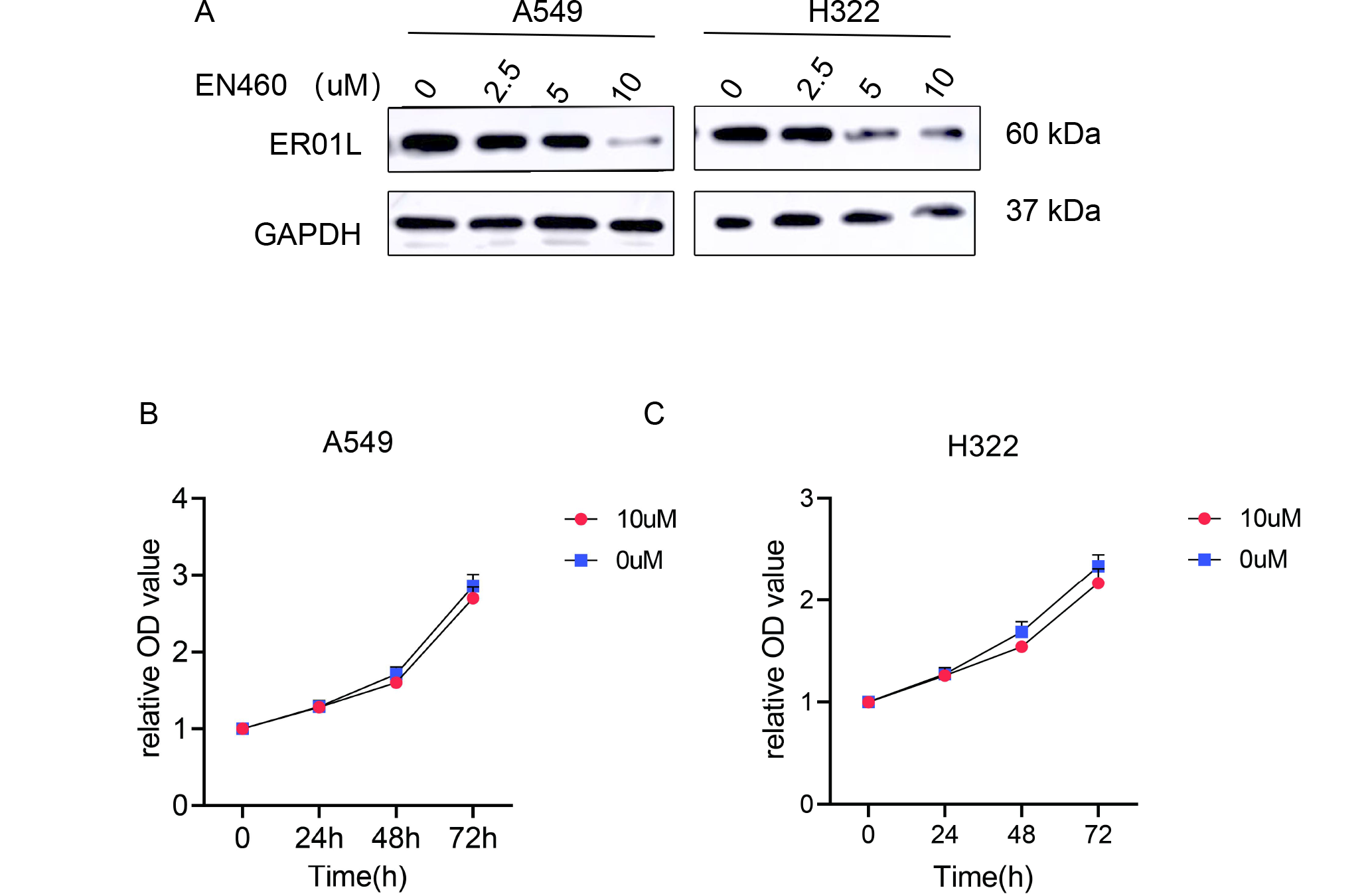

Supplement: Supplementary file 3 — figure S2 [file 41419_2020_3067_MOESM3_ESM.tif]

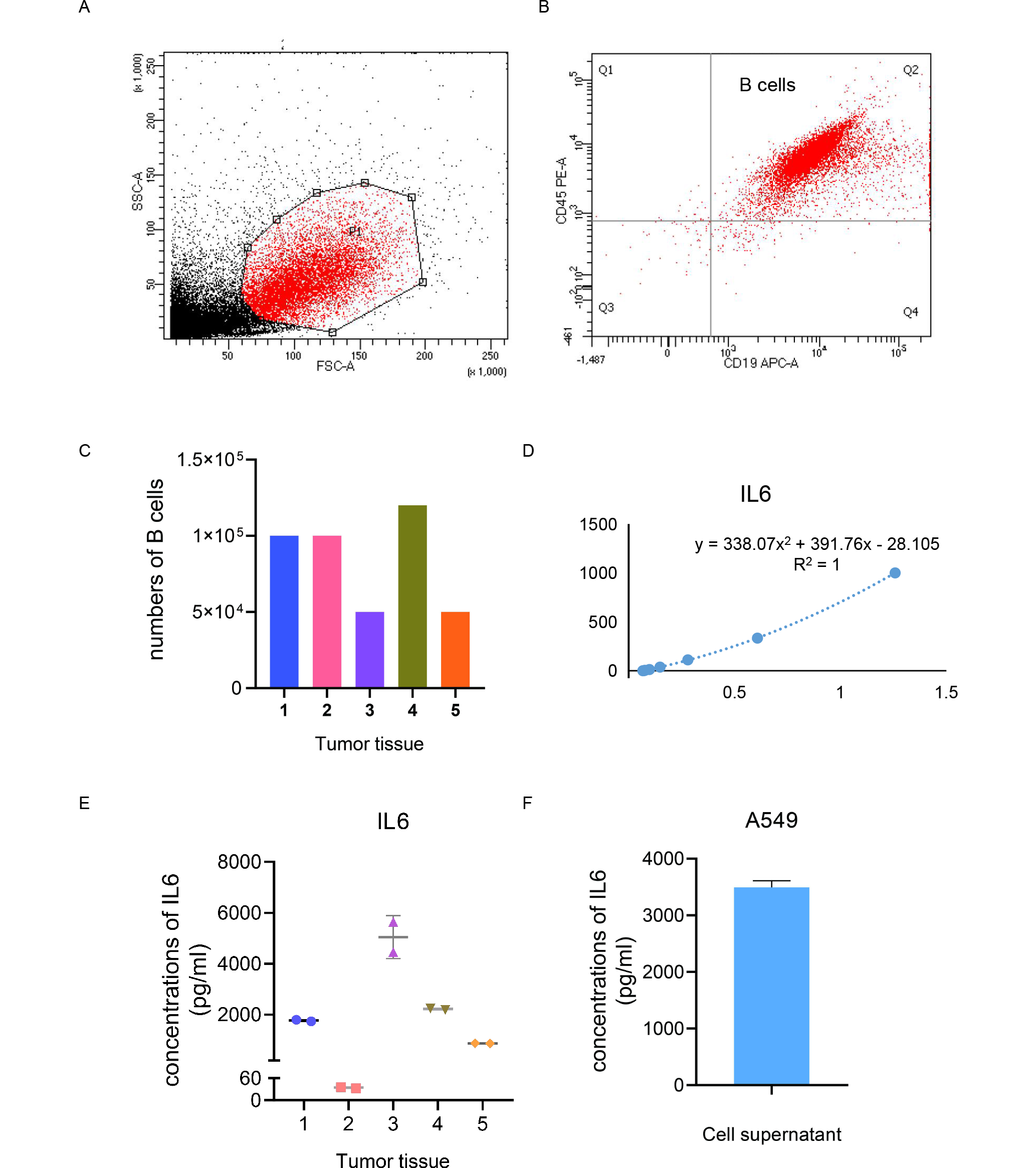

Supplement: Supplementary file 4 — figure S3 [file 41419_2020_3067_MOESM4_ESM.tif]
