# Supplementary material for: ERO1L promotes IL6/sIL6R signaling and regulates MUC16 expression to promote CA125 secretion and the metastasis of lung cancer cells
Source: Cell Death Dis. 2020 Oct 14;11(10):853. doi: 10.1038/s41419-020-03067-8 (PMC7560734; doi:10.1038/s41419-020-03067-8)
Supplement: Supplementary file 5 — supplementary [file 41419_2020_3067_MOESM5_ESM.docx]

## Supplementary Information

**ERO1L promotes IL6/sIL6R signaling and regulates MUC16 expression to promote CA125 secretion and the metastasis of lung cancer cells**

Yuanyuan lei^1^, Ruochuan Zang^1^, Zhiliang Lu^1^, Guochao Zhang^1^, Jianbing Huang^1^, Chengming Liu^1^, Zhanyu Wang^1^, Shuangshuang Mao^1^, Yun Che^1^, Xinfeng Wang^1^, Sufei Zheng^1^, Lingling Fang^1^, Nan Sun^1*^, Jie He^1*^

## Supplemental Data

Table S1 the top twenty genes most significantly associated with overall survival

| Gene Symbol | Gene ID | P value (Survival OS) |
| --- | --- | --- |
| [DKK1](http://gepia.cancer-pku.cn/detail.php?gene=DKK1) | ENSG00000107984.9 | 5.83e-8 |
| ERO1L | ENSG00000197930.12 | 4.17e-7 |
| [STEAP1](http://gepia.cancer-pku.cn/detail.php?gene=STEAP1) | ENSG00000164647.8 | 4.94e-7 |
| [INPP5J](http://gepia.cancer-pku.cn/detail.php?gene=INPP5J) | ENSG00000185133.13 | 2.27e-6 |
| [ANLN](http://gepia.cancer-pku.cn/detail.php?gene=ANLN) | ENSG00000011426.10 | 2.60e-6 |
| [RP11-462L8.1](http://gepia.cancer-pku.cn/detail.php?gene=RP11-462L8.1) | ENSG00000229656.6 | 2.97e-6 |
| [CASC5](http://gepia.cancer-pku.cn/detail.php?gene=CASC5) | ENSG00000137812.19 | 3.01e-6 |
| [FAM207BP](http://gepia.cancer-pku.cn/detail.php?gene=FAM207BP) | ENSG00000228797.2 | 3.23e-6 |
| [RP4-803A2.2](http://gepia.cancer-pku.cn/detail.php?gene=RP4-803A2.2) | ENSG00000239670.1 | 3.88e-6 |
| [S100A10](http://gepia.cancer-pku.cn/detail.php?gene=S100A10) | ENSG00000197747.8 | 4.88e-6 |
| [DYNLL1](http://gepia.cancer-pku.cn/detail.php?gene=DYNLL1)  [UBE2V2](http://gepia.cancer-pku.cn/detail.php?gene=UBE2V2)  [PKMYT1](http://gepia.cancer-pku.cn/detail.php?gene=PKMYT1) | ENSG00000088986.10  ENSG00000169139.11  ENSG00000127564.16 | 5.95e-6  7.38e-6 8.73e-6 |
| [HMMR](http://gepia.cancer-pku.cn/detail.php?gene=HMMR) | ENSG00000072571.19 | 8.82e-6 |
| [PSMA1](http://gepia.cancer-pku.cn/detail.php?gene=PSMA1) | ENSG00000129084.17 | 8.96e-6 |
| [CENPU](http://gepia.cancer-pku.cn/detail.php?gene=CENPU) | ENSG00000151725.11 | 9.28e-6 |
| [TFAP2A](http://gepia.cancer-pku.cn/detail.php?gene=TFAP2A)  [C1QTNF6](http://gepia.cancer-pku.cn/detail.php?gene=C1QTNF6)  [PSMC6](http://gepia.cancer-pku.cn/detail.php?gene=PSMC6) | ENSG00000137203.10  ENSG00000133466.13  ENSG00000100519.11 | 9.67e-6  1.22e-5  1.22e-5 |
| [MRPL13](http://gepia.cancer-pku.cn/detail.php?gene=MRPL13) | ENSG00000172172.7 | 1.24e-5 |

OS: Overall Survival

Group Cutoff: Median

Table S2 Clinical information of five lung cancer patients used to extract B cells

| ID | Gender | Age | Pathology | Tumor size | Stage |
| --- | --- | --- | --- | --- | --- |
| 1 | female | 40 | Lung adenocarcinoma | 0.8 | pT1bN0 |
| 2 | male | 60 | Lung squamous cell carcinoma | 0.2 | pT1aN0 |
| 3 | female | 63 | Lung adenocarcinoma | 1.6 | pT2aN0 |
| 4 | female | 47 | Lung adenocarcinoma | 0.7 | pT1bN0 |
| 5 | male | 74 | Lung adenocarcinoma | 0.5 | pT1bN0 |

Table S3 Possible binding sites of NF-kB and MUC16 promoter

| NC_000019.9:c9092468-9091969 Homo sapiens chromosome 19, GRCh37.p13 Primary Assembly | | | | MUC16 NM_024690 | | |
| --- | --- | --- | --- | --- | --- | --- |
|  |  |  |  |  |  |  |
| Name | Sequence | Position (0-based) | Strand | Score | p-value | E-value |
| [NF-kappaB (T00590)](https://biogrid-lasagna.engr.uconn.edu/lasagna_search/transfac_tfs/T00590.html) | ACGGGGTTTCTCCA | 89 | + | 131.69 | 0.00265 | 1.29 |
| [NF-kappaB (T00590)](https://biogrid-lasagna.engr.uconn.edu/lasagna_search/transfac_tfs/T00590.html) | CGGGGTTTCTCCA | 90 | + | 122.65 | 0.004 | 1.95 |
| [NF-kappaB (T00590)](https://biogrid-lasagna.engr.uconn.edu/lasagna_search/transfac_tfs/T00590.html) | GGGGTTTCTCCA | 91 | + | 120.6 | 0.004425 | 2.15 |
| [NF-kappaB (T00590)](https://biogrid-lasagna.engr.uconn.edu/lasagna_search/transfac_tfs/T00590.html) | TTGGGAGTTTGA | 120 | - | 107.37 | 0.00915 | 4.5 |

<https://biogrid-lasagna.engr.uconn.edu/lasagna_search/lasagna_search_varlen_ajax.php>

| Table S4 Primers used for real-time PCR | | |
| --- | --- | --- |
| GGene Symbol | Direction | Primer sequences |
| mERO1L | Forward | GGCTGGGGATTCTTGTTTGG |
|  | Reverse | AGTAACCACTAACCTGGCAGA |
| sMUC16 | Forward | TGTCTATGGGAAACAGCACTCAC |
|  | Reverse | GGATGAAAAAGGCAATGTTAAGC |
| MITGA10 | Forward | AACATCACCCACGCCTATTCC |
|  | Reverse | GTTGGTAGTCACCTAAGTGGC |
| MMARCKSL1 | Forward | CAAGGGTGAAGGGGAGTCG |
|  | Reverse | GACAGGCCGCTCAATTTGAAA |
| KRASD1 | Forward | AGCTGAGTATCCCGGCCAA |
|  | Reverse | CGATGGTAGGCGTGTAGGC |
| GRBMS3 | Forward | GGGGAACAGTTGAGTAAAACCA |
|  | Reverse | ACAATTTTTCCATACGGTTGGCA |
| SLCO4A1 | Forward | CTGCTCGCCCGTCTACATTG |
|  | Reverse | CCGAGGGTAACCAAGGATGG |
| VASH1 | Forward | GGTGGGCTACCTGTGGATG |
|  | Reverse | CACTCGGTATGGGGATCTTGG |
| CHL1 | Forward | ATGGAGCCGCTTTTACTTGGA |
|  | Reverse | GGCAACTTGGACTTTTGACTGT |
| CCDC80 | Forward | GACCCCGTTTCACTATGCTGT |
|  | Reverse | GGCGAGCTAGTCTCAACACG |
| IL6R | Forward | GACACTACTGGCGACGCACAT |
|  | Reverse | CACCCCATCCCTGACGACAAA |
| MUC16-C | Forward | TTCCAGAAGAGCAGCATGGG |
|  | Reverse | TCAGCTCCCAGTAAAGCTGC |
| IL6 | Forward | CCTCCAGAACAGATTTGAGAGTAGT |
|  | Reverse | GGGTCAGGGGTGGTTATTGC |

**Experimental Procedures**

**Cell culture**

A total of 4 lung adenocarcinoma cell lines A549, H322, H2009, H2030, and cell line 293T were selected for STR identification in this study. The four lung adenocarcinoma cell lines were all cultured in RPMI1640 medium containing 10% fetal bovine serum and 0.2% penicillin and streptomycin. For 293T cells, high-glucose DMEM medium supplemented with 10% fetal calf serum was used. All cells were cultured in a 37 ° C, 5% CO2 cell incubator.

**RNA extraction and quantitative real time PCR (qRT-PCR)**

In the whole process of RNA extraction, the tips, pipettes, EP tubes and solutions used should avoid RNase contamination. All operations should be performed in an RNase-free environment. Also pre-cool the centrifuge to 4 °C. Total RNA was isolated with the standard Trizol-based protocol (Invitrogen). For the extracted RNA, use Nanodrop instrument to detect concentration and purity, directly used for reverse transcription. Reverse transcription was performed by Revert-Aid First-Strand cDNA Synthesis kit (Thermo Scientific), the whole process should be carried out on ice to avoid RNase contamination. The reagents and consumables used in the experiment must be free of RNA contamination. RT-PCR was performed on an ABI 7900HT Real-Time PCR thermocycler (Life Technologies). After the PCR reaction was completed, the base and threshold of the amplification curve were manually set. Export the data results and calculate the average of each group of Ct. Data analysis using 2−ΔΔC method.

**Western blot**

Protein were extracted with 1×RIPA lysate plus 1% volume of protease phosphoric acid inhibitor (100 ×).The kit for measuring protein concentration was Pierce BCA protein assay kit (Thermo Fisher).After measuring the concentration, add 1/4 volume of 5× protein loading buffer according to the volume of the cell lysate, mix well after shaking, centrifuge in a metal bath, and boil at 100 °C for 30 minutes to fully denature the protein. Then, configure the appropriate concentration of PAGE gel, keep the same amount of protein in each lane. The separated protein was transferred to a PVDF membrane, blocked with 5% skim milk powder for 1 hour, and then incubated with primary antibody overnight. The primary antibody types used in the experiment were as follows: ERO1L(Abcam ab177156), ZO-1(CST 8193), GAPDH(CST 5174), E-cadherin(CST 3195), claudin-1(CST 13255), Vimentin(CST 5741), IL6R(Abcam ab128008,ab222101), NF-kB(CST 8242), p-NF-kB(CST 3033), p-IKKα/β(CST 2697T), and Tubulin (Abclonal). The blots were incubated with HRP-conjugated second antibody for two hours, then performed ECL chemiluminescence color development.

**Plasmids and reagents**

The overexpression vector of ERO1L and MUC16-C and the construction of the lentiviral plasmid of shRNA used in this study were completed by SyngenTech (Shanghai). The sequence was as follows:

MUC16(Human,14093-14507aa): 5’-ATGGGCAAGGGCTCAGCTACATTCAACTCCACCGAGGGGGTCCTTCAGCACCTGCTCAGACCCTTGTTCCAGAAGAGCAGCATGGGCCCCTTCTACTTGGGTTGCCAACTGATCTCCCTCAGGCCTGAGAAGGATGGGGCAGCCACTGGTGTGGACACCACCTGCACCTACCACCCTGACCCTGTGGGCCCCGGGCTGGACATACAGCAGCTTTACTGGGAGCTGAGTCAGCTGACCCATGGTGTCACCCAACTGGGCTTCTATGTCCTGGACAGGGATAGCCTCTTCATCAATGGCTATGCACCCCAGAATTTATCAATCCGGGGCGAGTACCAGATAAATTTCCACATTGTCAACTGGAACCTCAGTAATCCAGACCCCACATCCTCAGAGTACATCACCCTGCTGAGGGACATCCAGGACAAGGTCACCACACTCTACAAAGGCAGTCAACTACATGACACATTCCGCTTCTGCCTGGTCACCAACTTGACGATGGACTCCGTGTTGGTCACTGTCAAGGCATTGTTCTCCTCCAATTTGGACCCCAGCCTGGTGGAGCAAGTCTTTCTAGATAAGACCCTGAATGCCTCATTCCATTGGCTGGGCTCCACCTACCAGTTGGTGGACATCCATGTGACAGAAATGGAGTCATCAGTTTATCAACCAACAAGCAGCTCCAGCACCCAGCACTTCTACCTGAATTTCACCATCACCAACCTACCATATTCCCAGGACAAAGCCCAGCCAGGCACCACCAATTACCAGAGGAACAAAAGGAATATTGAGGATGCGCTCAACCAACTCTTCCGAAACAGCAGCATCAAGAGTTATTTTTCTGACTGTCAAGTTTCAACATTCAGGTCTGTCCCCAACAGGCACCACACCGGGGTGGACTCCCTGTGTAACTTCTCGCCACTGGCTCGGAGAGTAGACAGAGTTGCCATCTATGAGGAATTTCTGCGGATGACCCGGAATGGTACCCAGCTGCAGAACTTCACCCTGGACAGGAGCAGTGTCCTTGTGGATGGGTATTCTCCCAACAGAAATGAGCCCTTAACTGGGAATTCTGACCTTCCCTTCTGGGCTGTCATCCTCATCGGCTTGGCAGGACTCCTGGGAGTCATCACATGCCTGATCTGCGGTGTCCTGGTGACCACCCGCCGGCGGAAGAAGGAAGGAGAATACAACGTCCAGCAACAGTGCCCAGGCTACTACCAGTCACACCTAGACCTGGAGGATCTGCAATGA-3’

The shRNA sequences used for screening are as follows:

| Table S5 shRNA target sequences for ERO1L | | |
| --- | --- | --- |
| Gene Symbol |  | shRNA |
| ERO1L | sh1 | 5’-GGATGATTGTACCTGTGATGT CGAAACATCACAGGTACAATCATCC-3’ |
|  | sh2 | 5’-GGGCTTTATCCAAAGTGTTAC CGAAGTAACACTTTGGATAAAGCCC-3’ |
|  | sh3 | 5’-GGGTTGCTGGATTTAGCAAAT CGAAATTTGCTAAATCCAGCAACCC-3’ |

**Lentivirus packaging**

A: Plate: Digest the cells (wash once with PBS before digestion), add about 7ml culture medium (10% FBS in DMEM) and mix by pipetting. Add the cell suspension to the six-well plate, 1ml per well, mix by shaking Incubator;

B: Transfection: Take a six-well plate, add OPI-MEM, 1ml per well, add target plasmid 500ng in sequence, and package the plasmids PLP1 500ng, PLP2 500ng, and PLP3 500ng). Controls were labeled as shRNA scramble or LV-vector. Add 6ul lipo3000 to each well, mix by pipetting gently and incubate at room temperature for 15-20 minutes;

C: Change the medium: After 6-8 hours, use DMEM containing 30% FBS to change the medium. Add 2ml of medium to each well. Use gentle methods to avoid blowing the cells up;

D: Virus collection: collect the virus solution 48 hours after changing the solution, put the supernatant into a 2ml centrifuge tube, and directly infect the plated cells (50% confluence) or seal at -80 °C;

**Transcriptome sequencing**

The transcriptome sequencing was completed by Beijing Novogene. RNA samples were from stably transfected cell lines A549 and H322. After the library construction was qualified, different libraries were pooled according to the requirements of effective concentration and target offline data volume for Illumina sequencing. The basic principle of sequencing was sequencing by synthesis. The analysis process after obtaining the data includes quality control, comparison, quantification, significant difference analysis, and function enrichment. Selection of differential genes by screening standard fold change≥2; P<0.05.

IHC

The tissue chip was baked in an incubator at 80 ℃ for 2 hours, and then gradually dewaxed and hydrated with xylene and alcohol. Antigens were processed by microwave repair. After washing 3 times with PBS buffer, drop goat serum on tissue slides to cover the whole. Incubate the tissue on the chip for 30 minutes at room temperature, taking care to prevent the surrounding tissue from drying out. After the goat serum was blocked, shake off the excess serum and add the diluted primary antibody ERO1L (Abcam ab177156) to the tissue core, incubated at 4 ° C overnight. After incubating at room temperature for half an hour the next day, the secondary antibody was incubated and finally developed by BCA method.

ELISA

The kit for detecting IL6R, IL6 and CA125 were obtained from Raybiotech. For cell supernatant, centrifuge with 1000g for 20min, then store in refrigerator at -80° C for future use. For serum, first centrifuge with 1000g for 20 minutes, then dilute to a certain multiple. Set the standard sample hole and the sample hole to be tested and the blank control hole, and add 100ul each hole, and make two duplicate holes for each, incubate for 1 hour at 37℃ ,and add detection A, detection B, substance and stop solution according to the instructions given by the manufacturer. Finally, run the microplate reader and conduct measurement at 450nm immediately.

**Immunofluorescence**

The cells with good growth state and confluency of 80% were digested according to the conventional method using trypsin enzyme to prepare a uniform cell suspension and counted, and the cells were diluted to 50,000 cells/ml, and 100ul of cell suspension was taken. Add to 8-well chamber slides (Biolex) for 24 hours. Then, cells were fixed by 4% paraformaldehyde, ruptured by 0.3% Triton X-100 for 5 min, blocked by 5% BSA for half an hour, and then the corresponding primary antibody was added overnight. The primary antibody used in this study was CA125(Abcam ab1107), E-cadheirn (CST 3195T), claudin-1 (CST 13995), and ZO-1 (CST 8193). According to the different species of the primary antibody, prepare the secondary antibody with 5% BSA solution, add 100ul of the second anti-solution solution to each well, and store at room temperature for 1 hour in the dark. After DAPI (ROEY）staining, it was observed under an inverted fluorescence microscope in a dark room and photographed.

**Chromatin immunoprecipitation assay (Chip)**

Chip assay was performed using Simple Chip Enzymatic Chromatin Immunoprecipitation kit (9003, CST) according to manufacturer’s instructions. Briefly, collect appropriate amounts of ERO1L knocking down cell line A549 and control cell lines. Perform nuclear processing and chromatin cutting. A cross-linked chromatin fragment containing 5-10ug of DNA was incubated with KLF4 antibody for overnight at 4℃. Finally, the chromatin was eluted from the magnetic beads, and the DNA was extracted from the spinnator. The obtained DNA was used for subsequent PCR quantitative identification to calculate the enrichment efficiency. Specific primers specific to the MUC16-C promoter region were required. (Forward1: AGCCTGGTTCCTGGTTTCTAA; Reverse1: AGCCTGGTTCCTGGTTTCTAA; Forward2: GCCTGGTTCCTGGTTTCTAA; Reverse2: TGATCTCAATTCTTCCCTTCAAA).

**Microarray analysis of cell supernatant**

The experimental chip was purchased from raybiotech (guangzhou). Different treatment groups were plated according to the same number of cells, so that the confluency of the cells after adherence could reach 80%, and then the old medium was removed and changed to serum-free medium for 48 h. Add 100 µL of sample dilution to each well, and incubate for 1h at room temperature on a shaker to block the quantitative antibody chip. Then remove the buffer from each well, add 80μL of sample to the well, and incubate at 4°C overnight (samples are concentrated 8 times for loading). The next day, the slides were washed with a Thermo Scientific Well wash Versa chip washer, after which 80μL of detection antibody was added to each well, and incubated on a RT shaker for 2 hours. Wells were washed again and 80 µL of Cy3-streptavidin were added to each well. Then, cover the glass with aluminum foil and incubate in the dark, and incubate for 1 hour on a RT shaker. Use a laser scanner to collect fluorescence signals after the last washing.

**B cell extraction and isolation**

Tumor Dissociation Kit was from Miltenyi Biotec (Order no. 130-095-929). The dissociation of tumor tissue and the preparation of single-cell suspension were done as the procedures shown in the instructions. Cut the tumor into small pieces of 2–4 mm and transfer the tissue pieces into the gentleMACS C Tube containing the enzyme mix for the next operation. After the tissue was digested into a single cell suspension, CD19 microbeads (Miltenyi Biotec, Order no. 130-050-301) were used for B cell sorting. After sorting, the purity of B cell sorting was detected by flow cytometry. The selected antibodies were CD45 (Biolegend 103106) and CD19 (Biolegend 115511). The corresponding isotype antibodies were PE Rat lgG2b (Biolegend 400607) and APC Rat lgG2a (Biolegend 400511). The sorted B cells were placed in 1ml medium (procell CM-H030) and cultured for 48h, preparing for the next detection of IL6 in the cell supernatant by ELISA.

**Mouse tail vein injection lung colonization**

In this study, an experimental tumor metastasis animal model was used. NOD/SCID mice were purchased from Beijing Huakangkang Technology company. A total of 50 NOD/SCID mice, 4-5 weeks old, weighing between 17 g and 20 g, were raised in the heart center of the Chinese Academy of Medical Sciences. Mice were placed at a constant temperature between 25 °C and 27 °C and a constant humidity of 45% to 50%. While the NOD/SCID mice were adapted to the experiment environment, stable cell lines with ERO1L knocking down or overexpressing and respective control cell lines were expanded in vitro. When the degree of fusion of the cells in the culture dish reached 80 - 90% or more, the cells were digested with trypsin, a single cell suspension was prepared using the whole medium, and the cell concentration was adjusted to 10 ^7^ /ml using a cell counter. The cell suspension after completion of the counting was placed on ice and prepared to inject tumor cells into the tail vein of the mouse. During the injection process, the cells should be injected slowly and uniformly, not too fast, otherwise the mice were prone to heart failure. Within 48 hours after the tail vein injection, closely observe the state of the mouse. After 8 weeks, the mice were sacrificed by CO2 anesthesia, the lung tissue was completely dissected, and the blood on the lung tissue was washed with PBS buffer. The picric acid was fixed, photographed and embedded in paraffin and sectioned.
